# Violence rate dropped during a shift to individualized patient-oriented care in a high security forensic psychiatric ward

**DOI:** 10.1186/s12888-020-02524-0

**Published:** 2020-05-05

**Authors:** Ragnar Urheim, Tom Palmstierna, Knut Rypdal, Rolf Gjestad, Mette Senneseth, Arnstein Mykletun

**Affiliations:** 1 Norwegian Institute of Public Health, Department for Mental Health and Suicide, Oslo, Norway; 2 Social and Forensic Psychiatry Program, Stockholm Centre for Psychiatric Research and Education, Karolinska Institutet, Stockholm, Sweden; 3 Centre for Research and Education in Forensic Psychiatry, Haukeland University Hospital, Bergen, Norway

**Keywords:** Forensic psychiatry, Violence, Situational variables, Individualized care, Organisation

## Abstract

**Background:**

Contextual variables such as staff characteristics, treatment programs, assessment routines and administrative structures are found to influence patient violence rates in psychiatric forensic wards. The possible effects of current developments in treatment philosophy emphasizing patientsˈ perspective and treatment involvement upon violence rate have not yet been examined. The aim of this paper is to analyse associations between such developments and the occurrence of violent incidents among patients in a high security forensic psychiatric ward.

**Methods:**

During a 17-year period with stable ward conditions, incidents of violence were systematically collected together with diagnostic, risk assessment and demographic patient characteristics. Changes in care- and organizational related variables such as nursing staff characteristics, treatment and management routines were collected. Multilevel modelling was applied to estimate the relationship between these variables and changes in violent incidents.

**Results:**

A substantial decline in the occurrence of violent incidents paralleled with changes in the ward during the middle phase of the study period. Most of the changes, such as implementation of new treatment and care routines and an increased proportion of female staff and higher education levels, were significantly related to a decrease in the occurrence of violent incidents in the ward.

**Conclusions:**

Findings in this study suggest that an increase in individualized, patient-oriented care strategies, delivered by well-educated nursing staff with an equally balanced gender distribution contribute to a low level of violence.

## Background

Socio-ecological perspectives on human violence address individual as well as a wide range of contextual variables as causal factors [1]. Psychiatric patients have an increased violence rate, explained by individual factors [2–4] but also related to poor environmental living conditions. Violence inside psychiatric wards is reported more frequently than outside such settings [5] and is considered a serious milieu-problem for patients as well as staff. In addition, such behaviour poses challenges with regard to treatment. Literature reviews address a wide range of environmental and situational variables related to institutional violence [6–8], although the scarcity of research is also expressed [6]. Ward culture is considered to be an important violence-related factor [9] but the impact of care and a treatment philosophy emphasizing patientsˈ perspective and involvement introduced over the past decades [10, 11] has not yet been examined.

### Factors associated with institutional violence rate

Variations in ward settings such as characteristics of nursing staff are related to altered violence rate. A three-fold increase in violent incidents in an acute psychiatric ward was strongly associated with a considerable decline in permanent nursing staff [12]. Further, a higher proportion of female and educated nursing staff was related to lower violence rate in institutions for psychiatric patients with substance abuse [13]. A ward staffed only by women had no incidents, while a similar ward, traditionally staffed with mostly male had a usual high violence rate [14].

Studies of psychosocial measures, such as transition to an environment with improved communication routines and problem solving options [15] and introduction of collaboration routines between staff and patients about individual violence risk [16], have similarly reported significant reduced rates of institutional violence. Debriefing routines after staff exposure to patient aggression have also been found to contribute to declining violence rates [17]. But, studies of outcome of involving patients, such as shared staff and patient review of violent incidences (e.g. encouraging mutual perspectives on facilitating individual problem solving) is lacking.

The introduction of violence risk assessment procedures is also found to decrease levels of inpatient aggression; possibly by contributing to more individually adjusted proactive violence management [16, 18, 19]. These studies describe increased involvement of nursing staff as well as patients in treatment issues. This may facilitate patient-related perspectives and contribute to more individualized care.

Monotony and passivity can be a prominent institutional feature and violence is found to be more likely to occur in unstructured settings. The implementation of patient activity programs may give patients an opportunity to develop and use pro-social abilities [9, 20].

The general restrictive character in forensic settings, important for security, may also contribute to increased violence risks by measures that can be experienced as provocative [21–24]. However, we lack information about effects of specific conditions contributing to restricted autonomy such as opportunities for unescorted leave, exposure to sedating antipsychotic medication and expression of patient rights in legislation. Such aspects of patient care may influence the relation between restrictive versus individualized patient-oriented ward aspects. Ward administration is also relevant to the risk of violence [25] and patient turnover rate may indicate an active and offensive ward management with a potential impact upon quality of patient care.

Implementation of variables described in the studies mentioned above, affecting the degree of individualized patient-oriented care, may thus influence violence levels. The impact of such variables may be studied by evaluating interventions or unplanned environmental change [26]. Findings of such studies include increased violence rates after a decline in the number of permanent and experienced nursing staff [12], after the introduction of post-incident briefings for staff [17], psychosocial intervention in a prison ward [15], ward organizational changes [25], the implementation of risk assessment routines [18, 19] or staff-patient cooperation on the identification of warning signals [16].

### Historical background for the present study

The present study relies upon a previous study of the changes that took place during the years 1989–2006 in a high security forensic psychiatric ward in Norway. This study described a change process characterized by three different phases [27]. The first, from the time the ward opened in 1989 until 1994, was cautious and restrictive, with emphasis upon risk management at the expense of treatment ambitions. A more dynamic phase from 1995 to 1999 was characterized by changed routines, increased patient flow, professional innovations and more offensive treatment ambitions. This meant initiation of improved patient activity program, implementation of structured dialogues between staff and patients after violent incidents and of individual risk assessment procedures accentuating dynamic factors leading to less coercive risk management. Educational level and female / male proportion of the nursing staff increased by over 60% over a few years. New legislation, emphasizing patient rights were was introduced and influenced professional discussions. From 2000 to 2006, these changes were maintained and further developed, such as more dynamic risk assessment.

### Study aim

The present study aims to examine the relationships between changes in individualized patient-oriented care and patient violence rate.

## Methods

### Setting and sample

The site for the study is a 10-bed high security forensic psychiatric ward, covering an area of about one million inhabitants in South-Western Norway, providing services for the most violent psychiatric patients in the area. The study period was characterized by a stable ward mandate, obligations, resources and intake area, but also changes which may have influenced violence levels. In this paper, we will analyse these associations.

The patient population consists of all 55 patients admitted during the study period with a stay of more than 3 months, seven women and 48 men. The mean age of the patients was 34.8 years, *SD =* 9.3. The mean length of stay during the whole study period was 1119 days and the median length of stay 470 days. The mean and median length of stay for patients hospitalized over the years 1990–94 was 804/434 days, over the years 1995–99 it was 1054/559 days and over the years 2000–06 it was 1183/470 days. The mean length of stay increased over the phases.

Comorbid diagnoses, according to the ICD-10 [28] were the main rule. Among the 26 patients with schizophrenia and the 21 with other psychotic disorders, 33 also had a personality disorder, 21 of them dissocial. All patients were routinely assessed for risk of violence with Historical, Clinical, Risk-20 scale (HCR-20) and Psychopathic Checklist-Revised (PCL-R) (patients admitted before 1997 were assessed retrospectively) [29, 30]. The mean score on the HCR-20 scale (assessed 3 months after admission) was 25.1 and on the PCL-R 19.9. During the study period from 1990 to 2006, the studied sample of observations was 3594 violent incidents from 48 of these 55 patients.

### Description of variables

#### Violence data

Incidents were recorded with the Staff Observational Aggression Scale (SOAS/SOAS-R) [31, 32] recorded by the staff member exposed and/or witnessing, as soon as possible. The most severe incidents (> 8 on the SOAS-R severity scale) were selected for further analysis [12, 15]. Outcome measures in the analysis were annual and monthly rates, calculated from the incidents per occupied bed for years and months respectively. The annual rate was used to compare violence rates for different phases of the total period and to provide background information in trend graphs. The monthly rate formed the basis for describing violence trends and for examining the impacts of care and organizational variables.

#### Patient data

Annual group data of patient characteristics were mean age, ratio of female patients, percentage of patients with schizophrenia, dissocial and unstable personality disorder, and the mean score on HCR-20 and on PCL-R.

#### Contextual data

Ten care- and organization related variables from the earlier study [27] were found suitable for analysis. Of these, six involved interventions or other events:

- A procedure of shared staff and patient reviews of violent incidents implemented September 1994, encouraging structured discussions between assaulted staff and patients after violent incidents. The aim of this procedure was to improve staff-patient relations and support alternative problem solving and coping
- A mandatory patient activity program, with the purpose of structuring daily life, was instigated from 1995 but came to an end in 2001.
- A change in medication policy with gradually reduced use of sedating antipsychotic medication began in 1995, followed by the introduction of second generation antipsychotics in about 1997.
- A violence risk assessment procedure, applying the HCR-20, was introduced from 1997 and was further elaborated on after 2000 followed by increased participation from the entire nursing staff.
- Multi-disciplinary treatment plans and treatment meetings, involving coordinated plans and improvements in patient teams were implemented from 1998.
- New legislation, included the discussion of preliminary work which got started in 1998, raised important issues about patient rights. The law entered into force at the end of 1999.

A variable not present a given year was given the value 0 for this year. The variables *shared staff and patients review of violent incidents* and *mandatory activity program* were given the value 1 if they were present. The variables *changes in medication policy*, *violence risk assessment procedure*, *multi-disciplinary treatment plans and treatment meeting* and *new legislation* were given the value 1 if they were partly and the value 2 if they were fully present a given year.

Four variables, with a changing pattern during the years 1995–1999 describe the trends of annual values of:

- The proportion of weeks with unescorted leave of total weeks
- The patient turnover rate, i.e., the number of patients’ entries per year.
- The proportion of total nursing staff who are female.
- The proportion of health-educated staff, i.e., with health education of three or more years.

The staff variables include nursing personnel in direct interaction with patients, calculated from proportion per shift with these characteristics, in all shifts over 13 years.

These 10 variables are graphically presented, each against a background of the annual violence rate.

These variables were further combined into a new common variable, intended to give an overall measure of the ward development toward individualized patient-oriented care. Based on a principal component analysis the 10 variables were used in a weighted-factor score, representing the annual change in individualized patient-oriented care over the study period. To reduce the very high correlation between the variables of the proportion of female and health-educated staff, the female staff variable was regressed on the health education variable and the residual variance saved as a new variable. This new variable accounted for the unique variance not being statistically explained by the education level variable.

### Statistical analyses

SPSS version 23 was used for descriptive analyses, and correlations [33]. Data are clustered with 3594 violent incidents nested within 48 patients and Mplus 8.0.

was used for multilevel (ML) and single-level analysis with corrected standard errors when having clustered data [34, 35]. Incident and patient information may then be included at two different levels in the same statistical model. The sample size of 48 subjects at the between level in a multilevel model was considered as sufficient when analysing prediction models with only a few predictors at that level [36]. The focus is the incident level, and relations between patient- and contextual variables were entered at that level with corrections of standard errors in single level models and in multilevel models via cross level interactions (random effects on within level included at between level). The estimator was Maximum Likelihood with standard error corrections in case of skewness (MLR) [35, 37]. Multilevel modelling was used to estimate within- and between patient variations, giving including intra-class correlations (ICC) and the Design Effect (DE) [38]. DE values above 2 indicate non-ignorable data clustering. Then, a multilevel model explored whether the violence level was dependent on time. A standard random intercept random slope model analysed the additional information describing the baseline level and change over time of individual patients. The outcome variable, incidences of violence per time unit, is a count variable and the model was specified with Poisson regression. However, statistical significant dispersion (1.03, *p* < .001) and fit results (Poisson model: AIC was 10,002, BIC = 10,019, SABIC = 10,009; negative binomial model: AIC = 6886, BIC = 6902, SABIC = 6892) indicated the negative binomial regression as the preferable model. Model fit was evaluated with Akaike Information Criterion (AIC) the Bayesian Information Criterion (BIC) and Sample-adjusted BIC (SABIC) with lower values indicating a better model fit [39].

The overall care- and organizational factor (COF) was entered as a predictor of violence. Since the 10 variables increased over the years it should be strongly related to the time variable. Using both the overall factor and the time variable could therefore result in a high degree of multicollinearity and modelling problems. When this was the case, the time covariate would be left out of the model. The relation between the overall care- and organizational factor and violence was analysed for the entire study period, but also separated by the time intervals 1990–1994, 1994–1999 and 2000–2006. Additionally, the separate contextual variables were tested in simple regression models and in a multivariate model.

## Results

Forty-eight patients were involved in 3594 violent incidents. The aggregated total number of incidents per patient was 74.88 (SD = 127.86) with minimum one and a maximum of 526. At the month level the mean number of incidents was 1.89 (SD 3.17, Skewness = 4.11, kurtosis = 27.96). A multilevel model indicated differences between patients with considerable variation within patients (σ^2^_between_ = 4.42, *p* = .043; σ^2^_within_ = 7.84, *p* < .001; ICC = .36; DE = 15.75) indicated non-ignorable clustering. Seven patients (14.6% of total) accounted for 68.4% of the incidents. These seven patients were in the ward for a much longer period than the other patients were (142.43 vs 24.83 months, t = 5.85, *p* = .001) and thus the rates of violence were 2.75 (SD 1.37) versus 1.72 (SD 2.55), a difference not statistically significant (t = 1.04, *p* = .304).

### Reduction in violence

**Fig. 1:**
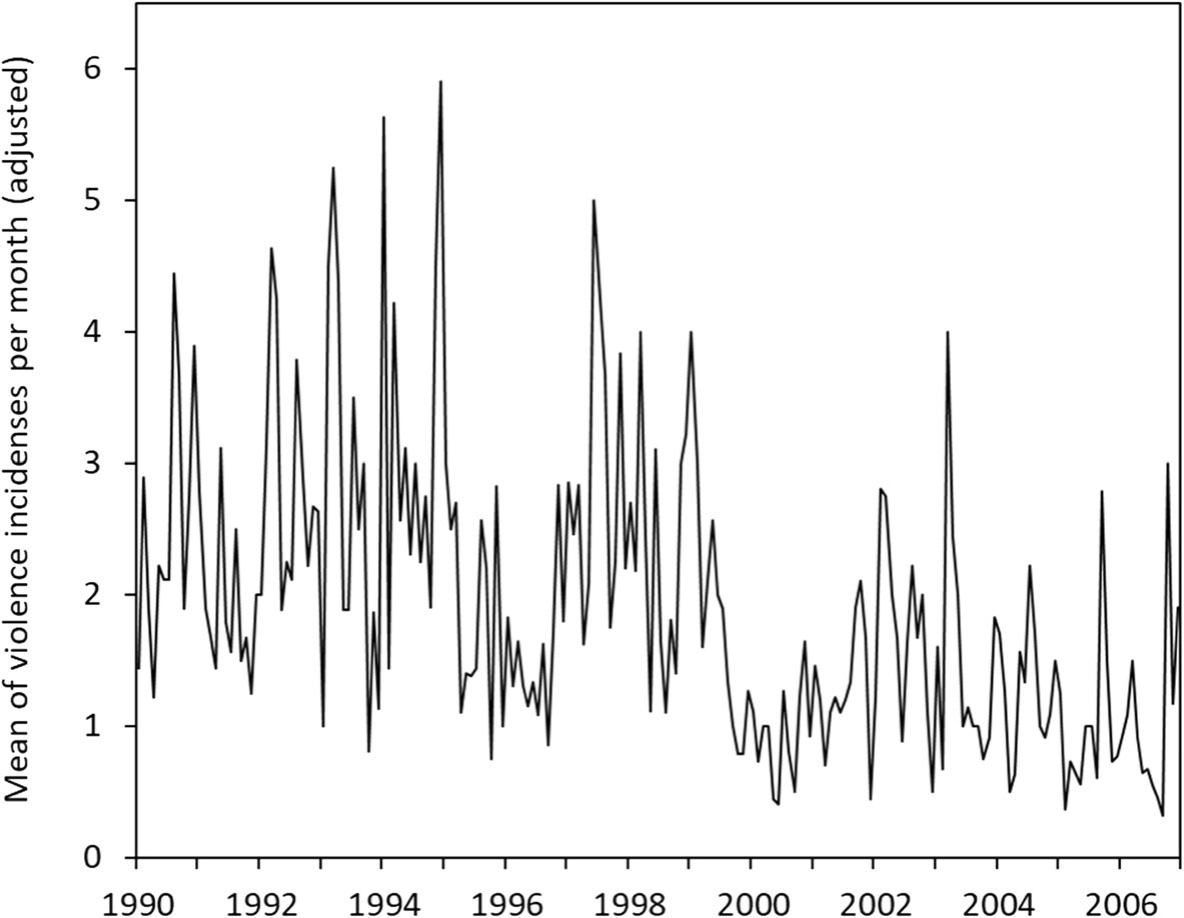
Violence rate. Legend: Development in the violence rate per month over 17 years. The count estimate is adjusted for the duration of stay in hospital department

The mean yearly rate of incidents per patient in the first phase (1990–1994) was 30.11 (95% CI: 21.63–38.61) and in the third phase (2000–2006) 13.95 (95% CI: 12.08–15.84), which shows a statistically significant reduction. The mean level in the first phase was estimated to be 2.64 (SD 3.60) and in the last phase 1.29 (SD 2.62), which gives a moderately sized effect (*d* = 0.44, based on a pooled within SD). The seven patients being present more and thus causing most incidents showed a stronger linear decrease in violence than the rest of the patients (0.06 versus 0.13 per year) using linear mixed analysis. However, as these are not qualitatively different from the other beyond quantitative different levels in violence rate at baseline, a better representation of data is the finding of a negative relation between baseline and change (covariance = − 0.16, *p* = .001). Most reduction was seen among the patients with highest level of incidents in the baseline phase. Figure 1 shows that violent incidents declined substantially over the 17 years for all patients, however, with considerable variation. A multilevel model showed no relation between time and violence at patient levels (b = 0.003, *p* = .457) or at the incident level (b = − 0.01, *p* = .067). However, a single-level model with corrected standard errors showed this time estimate to be statistically significant (*p* = .014).

The mean linear change in violence was found to be − 0.01 (*p* < .001; intercept = 1.45, *p* < .001), i.e., the incident rate decreased over time (predicted score = e^-0.011^ = 0.99, CI = 0.985–0.999). The slope variance describing individual differences in change was not found to be statistically significant (0.00, *p* = .948). The model fit was: AIC = 6777, BIC = 6805 and SABIC = 6790.

### The relationships between changes in care- and organizational variables and reduced violence rate

**Fig. 2:**
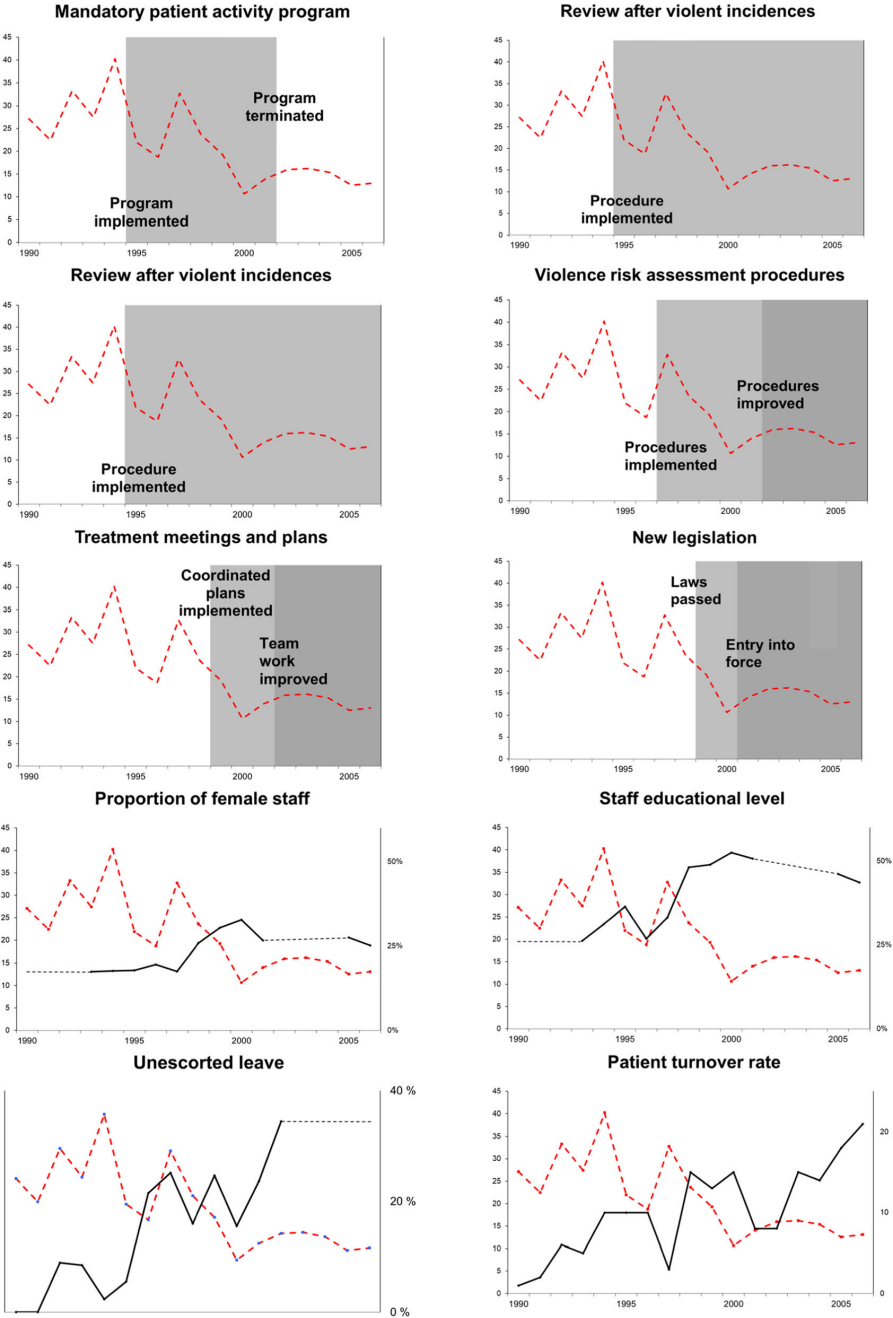
Care- and organizational variables. Legend: Annual values of care- and organization related variables against the annual violence rate (dotted line)

The 10 care- and organizational related variables coincided with a declining trend in annual violence rates (Fig. 2). All the variables changed or were implemented during the years of a decline of annual violence (1995–1999). Related to strong predictor correlations, none of these predictor variables were, however, found to be statistically significant in the multivariate model. Applying bivariate regression, fewer incidents per months were associated with seven of the 10 predictor variables: higher staff educational level (b = − 2.74, *p* = .034), higher proportion of female staff (b = − 5.49, *p* = .011), the implementation of shared staff and patient review after violent incidents (b = − 0.49, *p* = .027), multi-disciplinary treatment plans and meetings (b = − 0.33, *p* = .011), reduced use of sedative antipsychotic medication (b = − 0.32, *p* = .023), new legislation (b = − 0.32, *p* = .008) and a higher patient turnover rate (b = − 0.05, *p* = .009).

**Fig. 3:**
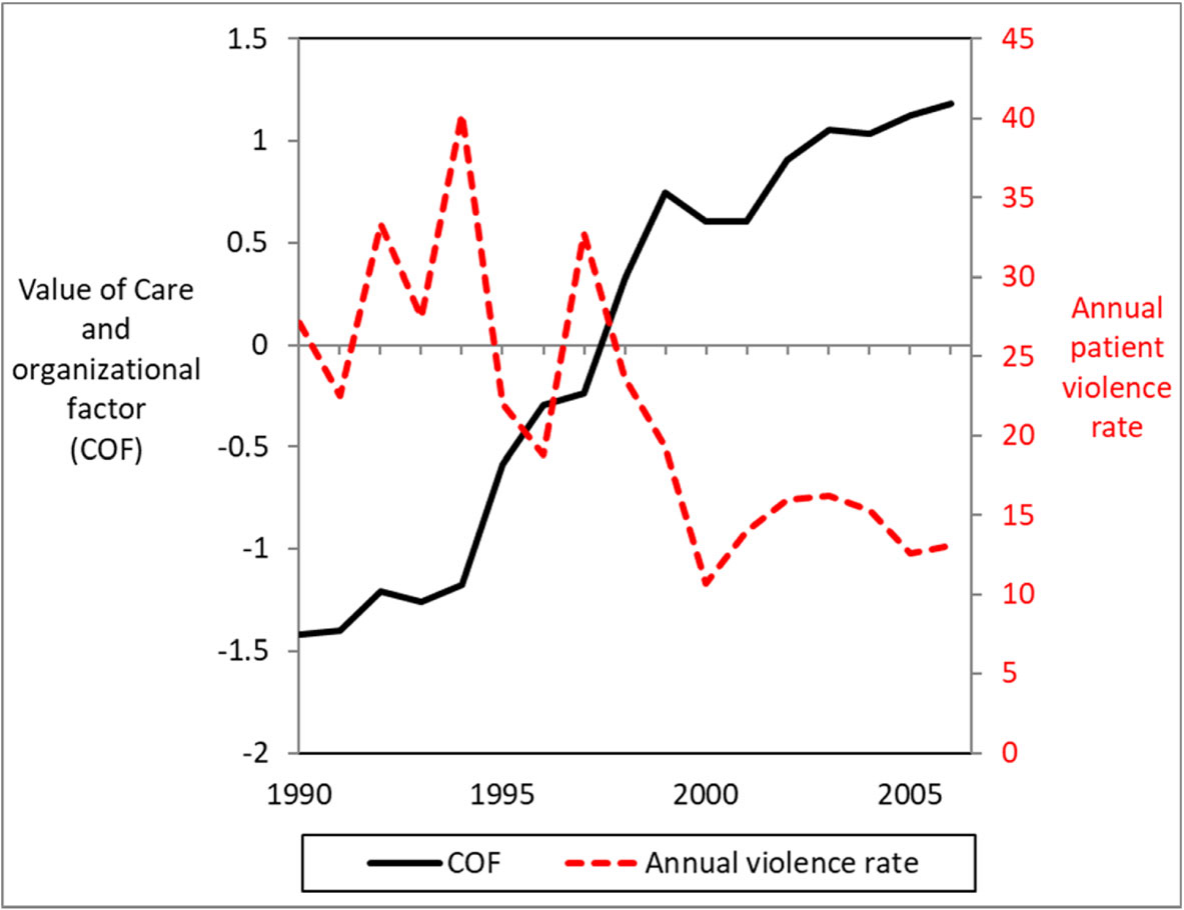
Care- and organizational factor. Legend: The level of the care- and organizational factor (COF), measured as a component COF factor score (solid line), over the period 1990–2006 compared to the annual violence rate (dotted line)

The weighted variables included in the COF factor were strongly related to time (r = .97, *p* < .001), presented in Fig. 3. The reduction in the level of violent incidents was found to be related to the linear increase in the overall care- and organizational factor (b = − 0.01, *p* = .049; predicted score = e^-0.007^ = 0.99, CI = 0.99–1.00). The linearity restriction was then removed. The incident level per month was now found to be more strongly related to the care- and organizational variables (b = − 0.30, *p* = .017, predicted score = e^-0.30^ = 0.74, CI = 0.56–0.92). A lower monthly violence rate was associated with higher levels of those variables.

Among the patient variables, the ratio of unstable personality disorder was positively related to violence (b = 1.36, *p* < .001). At patient level, however, the unstable personality disorder proportion did not change as a function of time (unstable personality disorder = 0.68, *p* = .240; interaction between unstable personality disorder and time: b = − 0.02, *p* = .801).

## Discussion

The occurrence of violent incidents decreased substantially on this forensic high security ward in the middle phase of the 17 years of observation. Changes in 10 care- and organizational variables took place during this decline phase. The increasing level of an overall factor, composed of the 10 variables, coincided with this decline in the rate of violence. Seven of 10 care- and organizational variables were significantly associated with violence levels, but due to collinearity, their relative impact could not be assessed. We will argue that the increase in the overall factor, strongly associated to the declining violence rate, relates to a shift towards individualized patient-oriented care. The care- and organizational variables may have affected this change process to varying degrees.

An important intervention may have been the shared staff and patient review after violent incidents (reflecting upon both staff and patients’ perspectives), which was implemented early in the declining trend in violent incidents. A similar procedure is recommended in NICE guidelines [40]. This routine may contribute to improved understanding of warning signals and precipitations and may have the potential to influence staff-patient interactions and relations. Positive relationships with staff is highlighted as essential for personal recovery in forensic patients in several studies [41–43]. Forensic patients report that feeling safe and understood, and to have trust in staff is a crucial part of their recovery [41, 43].

Among nursing staff characteristics, the increased proportion of female staff may have played an important role. The proportion of female staff increased in a couple of years parallel to reduced violence rate. Corresponding findings are reported in previous studies [14, 44]. Explanations suggested were same-sex aggression [44] and that use of male nursing staff to control violence may represent a self- fulfilling prophecy [14]. The increase in health-educated staff also seems to be an important factor in our study. However, the literature presents inconsistent findings in this topic [41] and further investigation of the importance of such a variable is needed.

Restrictions and loss of freedom, leading to every-day frustrations, may increase violence risk [45]. Opportunities for unescorted leave, increasing patient autonomy, reduced use of sedating antipsychotic medication and implementation of new legislation, increasing in patients’ rights, may have contributed to less perceived coercion. These variables were related to the level of violence, but in the case of unescorted leave, the link was not significant. This finding is interesting, as unescorted leave would seem as a measure of freedom and one might expect the opposite finding. However, from these findings, it seems more important to have less restrictions inside the ward, as well as increased patient rights in their treatment, to reduce violent incidents. Furthermore, escorted leave may also have enhanced connectedness and support from staff. Connectedness is emphasized as one of the essential recovery processes for patients with mental illness [46].

Patient turnover rate increased during the period. Higher violence risk is found among newly hospitalized patients [47], but our finding may suggest more ambitious and encouraging treatment and discharge plans, and thereby increased experience of being able to move forward in life. Implementation of multi-disciplinary treatment plans and treatment meetings may have reinforced this trend.

Some variables, contrary to expectations about possible impact upon violence rate, had a weak and non-significant relation. Violence risk assessment procedures could be expected to contribute to violence prevention [18]. Nursing staff were however not actively involved in such measures the first years following implementation. The importance of varied activities and meaningful life is emphasised in other studies [20], but the mandatory patient activity program was not related to violence rate. This was, however introduced as a group measure, rather than an opportunity for the individual patient, and the provocative aspects of this measure may have contributed to raise levels of conflict.

None of the individual patient characteristic examined, except unstable personality disorder, were related to violence. Patients with this diagnosis are overrepresented in forensic settings, and linked to high rates of aggression [48]. However, this variable cannot explain the violence decline, as the proportion of patients with unstable personality disorder did not change during the study period.

The overall care- and organizational factor overlaps with the wide range of contextual violence-related variables described in literature-reviews [6–8]. Additional aspects of the ward context, as staff-patient relations in risk and violence management are however also addressed. A possible impact of common staff and patient review after violent incidents, intended to take care of the aggressor-victim relation and to facilitate problem solving is not found in literature. The marked increase in the proportion of women in the nursing staff, previously rejected for security reasons, may also have contributed to risk management less dependent upon the use of force and thereby less provocative. This may have contributed to relational alternatives to the prevailing force-based approaches, tempting to use in difficult situations.

Safety-building practices, as cooperation about risk management, are found in wards with low levels of violence [16, 49]. Studies based upon patient and staff views also emphasise relations and individualized aspects of risk management in forensic settings. Relationships are highlighted as a fundamental part of recovery trajectories [41], therapeutic alliances are seen as crucial in risk assessment, providing personal information about the patient and contributing to inclusion and participation [50] and encouraging patient participation is considered important in avoiding and preventing violence [51].

Emphasis upon patient perspectives and cooperation is particularly interesting considering the recent recovery-approach in forensic psychiatry [52], promoting patient responsibility, shared decision making and self-determination [53]. There are similarities between the described ward change process and domains of recovery-oriented violence prevention strategies. Although not directly influenced by the recovery philosophy, the ward change which underlies this study may have common sources in the prevailing professional discussions toward the end of the twentieth century.

### Strengths and limitations

For reasons such as the discontinuity of nursing staff, a lack of reliable data and unstable clinical and administrative conditions, there is a shortage of long-term clinical studies on the impact of contextual factors. Such studies can be complicated by a limited time span, natural fluctuations in violence rates within institutions, the delayed effects of interventions or limited control of internal and external conditions. All these factors may weaken the validity and generalizability of outcomes.

In this study, a long series of reliable incident data, relevant clinical information and historical sources is available. Change in important ward characteristics in an otherwise stable setting provides features of a quasi-experimental design. The present study, examining the impact of care- and organizational variables upon inpatient violence, addresses the call for knowledge about the impact of contextual factors.

A rival hypothesis to the impact of a care and organizational factor, not examined in this study is that patients admitted later in the study period were less violent than the original patients were. In addition, the temporal clustering of change in care variables also limits the possibility of drawing strong conclusions about causality. The dissimilar form of the variables also excludes direct comparison of variables. Data sources are also limited. The sample of care- and organizational variables could have been more comprehensive, for example about organization and leadership [9, 25], the social climate [49] or the quality of staff-patient interaction or aggression management skills [54]. Some of the variables may be weakly related to central aspects of individualized patient-oriented care. To test the rival hypothesis of change in patient characteristics more exhaustively, we would have needed additional patient data, e.g., individual dynamic, fluctuating risk data, or data about patients before admittance and quality of preceding services delivered to the patients.

### Implications

The importance of the dimension of individualized care-orientation may contribute to the understanding of institutional violence and give some clues about intervention strategies. In addition to situational risk factors of institutional violence included in instruments such as Promoting Risk Intervention by Situational Management (PRISM) [55], this study address protective factors, such as patient perspective, staff-patient cooperation and thereby promoting more confident staff-patient relations.

The study’s findings are relevant with regard to further adaptation of the recovery-oriented limitations of this study. Care- and organizational variables must be included in larger controlled studies with a longitudinal design. Knowledge about contextual impact upon violence in clinical settings is requested, but also challenging due to the large range of potential environmentally confounding and dynamic variables. This study highlights the importance of an individual-oriented care dimension of such variables as well as the importance of mixed gender staffing and educational level of the nursing staff members. This pattern appeared after examining a multifaceted change process. Useful knowledge can be gained by studying long term change patterns in ward settings. Relevant clinical and milieu data is useful for the testing of short-term hypotheses as well as clinical decision making. Access to such variables may facilitate and inspire further case-studies based on a diversity of sources and methods [56, 57].

## Conclusions

The present study found that a shift towards individualized patient-oriented care, delivered by a well-educated nursing staff with a balanced gender proportion, was related to a reduced rate of violent incidents in a high secure ward. These findings are particularly important concerning the identification of factors that may reduce inpatient violence.

## Data Availability

Due to legal restrictions, the data cannot be made publicly available. The datasets used and analysed during the current study are available from the corresponding.

## Abbreviations

AIC: Akaike Information Criterion
BIC: Bayesian Information Criterion
COF: Care- and Organizational Factor
DE: Design Effect
HCR-20: Historical, Clinical, Risk-20 scale
ICC: Intra-Class Correlations
ML: Multilevel Analysis
MLR: Maximum Likelihood with Robust standard errors
PCL-R: Psychopathic Checklist-Revised
PRISM: Promoting Risk Intervention by Situational Management
SABIC: Sample-adjusted BIC
SOAS: Staff Observational Aggression Scale
SOAS-R: Staff Observational Aggression Scale-Revised
