# Correction to: Violence rate dropped during a shift to individualized patient-oriented care in a high security forensic psychiatric ward

**DOI:** 10.1186/s12888-020-03032-x

**Published:** 2021-01-15

**Authors:** Ragnar Urheim, Tom Palmstierna, Knut Rypdal, Rolf Gjestad, Mette Senneseth, Arnstein Mykletun

**Affiliations:** 1 Department for Mental Health and Suicide, Norwegian Institute of Public Health, Oslo, Norway; 2 Social and Forensic Psychiatry Program, Stockholm Centre for Psychiatric Research and Education, Karolinska Institutet, Stockholm, Sweden; 3 Centre for Research and Education in Forensic Psychiatry, Haukeland University Hospital, Bergen, Norway; 4 Western Norway University of Applied Sciences, Bergen, Norway

**Correction to: BMC Psychiatry 20, 200 (2020)**

**https://doi.org/10.1186/s12888-020-02524-0**

Following publication of the original article [1], the authors identified errors in the affiliations. The correct assigned affiliations to the authors are given below.

Ragnar Urheim3, Tom Palmstierna3,2, Knut Rypdal3, Rolf Gjestad3, Mette Senneseth3,4

3.Centre for Research and Education in Forensic Psychiatry, Haukeland University Hospital, Bergen, Norway

4.Western Norway University of Applied Sciences

The author group has been updated above and the original article [1] has been corrected.
